# Clostridium perfringens Bacteremia Secondary to Stercoral Colitis in a Patient With Advanced Parkinson’s Disease: A Rare Case Report

**DOI:** 10.7759/cureus.103471

**Published:** 2026-02-12

**Authors:** Nidhay Prakashkar, Jaskaran Singh, Shreya Muralidharan, Cheyenne Moffett, Sorabh Sharma

**Affiliations:** 1 Internal Medicine, B. J. Medical College, Ahmedabad, IND; 2 Internal Medicine, Government Medical College, Patiala, Patiala, IND; 3 Internal Medicine, Vydehi Institute of Medical Sciences and Research Centre, Bangalore, IND; 4 Diagnostic Radiology, University of Arizona College of Medicine, Tucson, Tucson, USA; 5 Internal Medicine, University of Arizona College of Medicine, Tucson, Tucson, USA

**Keywords:** atypical bactermia, bacteremia, clostridium perfringens, parkinsons disease, stercoral colitis

## Abstract

*Clostridium perfringens* infections are often associated with high mortality and typically result from soft tissue infections. Stercoral colitis, a complication of fecal infection, is more frequently linked to gram-negative bacteremia. We present a rare case of *C. perfringens* bacteremia caused by stercoral colitis. A 62-year-old woman with advanced Parkinson's disease, dementia, and immobility presented with altered mental status. Stroke was ruled out. Blood cultures grew *C. perfringens* without signs of soft tissue infection. A CT of the abdomen showed stercoral colitis with fecal impaction. She was treated with a seven-day course of intravenous metronidazole and an aggressive bowel regimen. She remained hemodynamically stable and was discharged in stable condition for outpatient follow-up. This case highlights stercoral colitis as a potential source of C. perfringens bacteremia even without perforation or soft tissue involvement. Early antibiotics, bowel decompression, and nutritional support can improve outcomes in high-risk patients.

## Introduction

*Clostridium perfringens*, an anaerobic gram-positive spore-forming bacillus, causes infections ranging from food poisoning to severe soft-tissue infections, such as gas gangrene. Bacteremia is associated with high mortality rates of 27-44% [1] due to severe complications, including massive intravascular hemolysis and septic shock [2,3]. Clostridial bacteremia is uncommon, accounting for 1-3% of invasive bacteremia in hospitalized patients [4]. Stercoral colitis, resulting from fecal impaction and mucosal ischemia [5], typically involves gram-negative bacteremia due to translocation of enteric flora [6]. We present a rare case of isolated *C. perfringens* bacteremia secondary to stercoral colitis in a patient with advanced Parkinson’s disease, demonstrating successful outcomes with early intervention [1,2] despite reported mortality rates of 27-44% [1].

## Case presentation

A 62-year-old woman with a complex medical history, including advanced Parkinson's disease, psychosis, audiovisual hallucinations, affective disorder, dementia, dysautonomia, REM sleep behavior disorder, failure to thrive, and weight loss due to dysphagia, presented to our facility with a code stroke. She showed an altered mental status and a questionable right facial droop, prompting urgent neurological assessment. Her baseline functional status included wheelchair dependence, requiring assistance with daily living activities, and moderate cognitive impairment from dementia. Vital signs were stable upon arrival. 

The differential diagnosis for her altered mental status included acute ischemic stroke, worsening Parkinson disease-related psychosis, dementia progression, infectious encephalopathy, metabolic or electrolyte imbalances, medication effects (e.g., from levodopa), or a seizure/post-ictal state. A neurological assessment and a brain MRI ruled out acute stroke, showing no infarction but revealing nonspecific extensive iron deposition in the bilateral basal ganglia, consistent with neurodegeneration associated with advanced Parkinson’s disease. 

Laboratory results obtained at the time of presentation are summarized in Table 1.

**Table 1:** Laboratory Investigations at Presentation Laboratory values were obtained at the time of presentation. Reference ranges represent standard adult values and may vary slightly by institution.

Parameter	Patient Value	Reference Range
White Blood Cell Count	6.4 × 10^9 /L	4.5 - 11.0 × 10^9 /L
Neutrophils	75%	40% - 60%
Lymphocytes	16.9%	20% - 40%
Hemoglobin	11.0 g/dL	13.5 - 17.5 g/dL (M) / 12.0 - 15.5 g/dL (F)
Platelet count	226 × 10^9 /L	150 - 450 × 10^9 /L
Creatinine	0.33 mg/dL	0.7 - 1.3 mg/dL (M) / 0.6 - 1.1 mg/dL (F)
Sodium	139 mmol/L	135 - 145 mmol/L
Potassium	3.8 mmol/L	3.5 - 5.0 mmol/L
Albumin	3.0 g/dL	3.5 - 5.5 g/dL

Urinalysis was negative, excluding urinary tract infection. Blood cultures obtained on admission from two separate sets revealed gram-positive rods, later identified as *Clostridium perfringens*. The patient exhibited no signs of soft tissue infections, gas gangrene, or cellulitis, with normal extremities.

**Figure 1:**
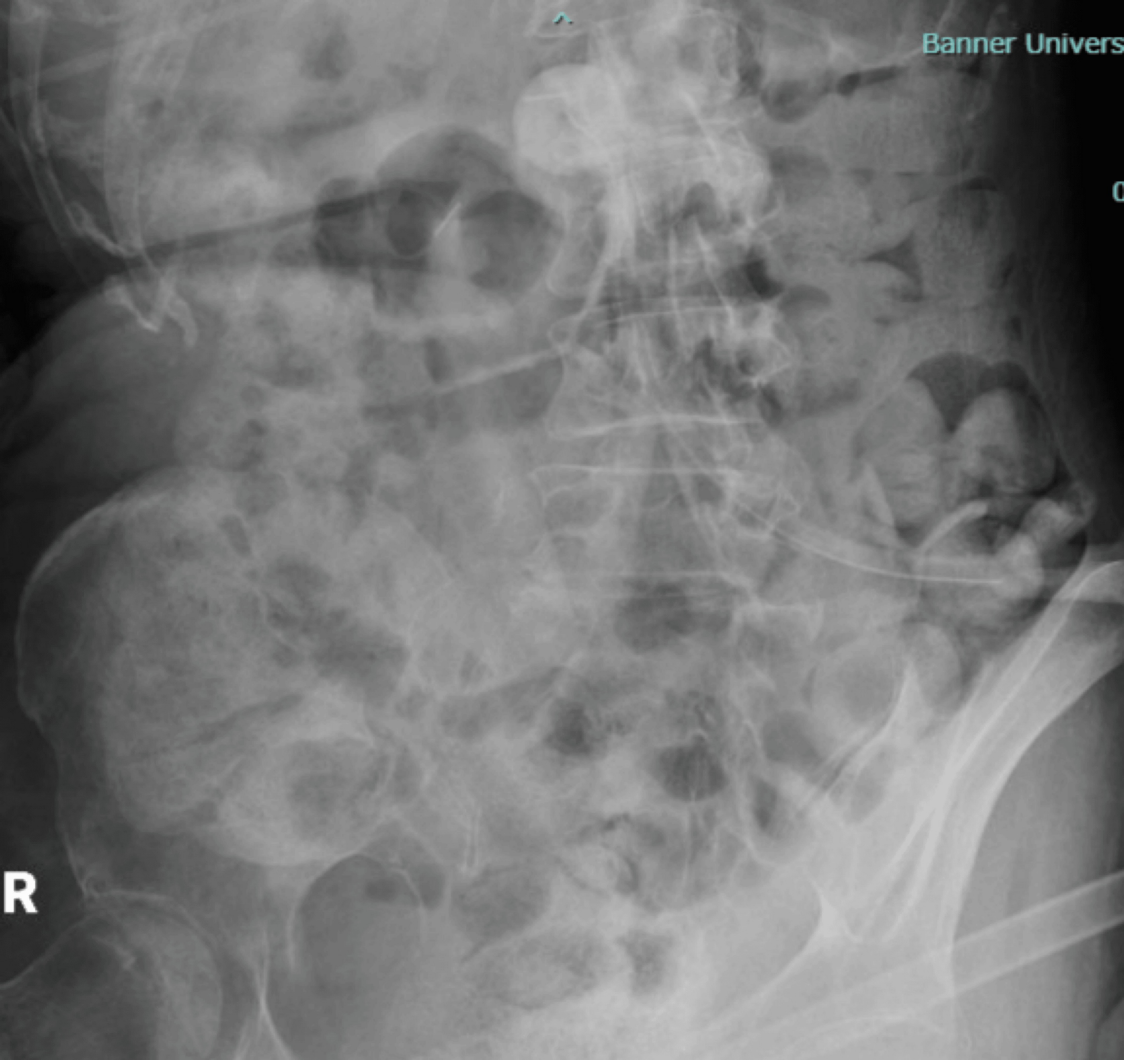
Erect Abdominal Radiograph Erect abdominal radiograph revealing diffuse colonic fecal loading, consistent with a heavy stool burden.

The high suspicion of stercoral colitis, considering advanced Parkinson’s, associated with decreased mobility, prompted further imaging. An erect abdominal radiograph revealed diffuse colonic fecal loading, consistent with a heavy stool burden (Figure 1).

**Figure 2:**
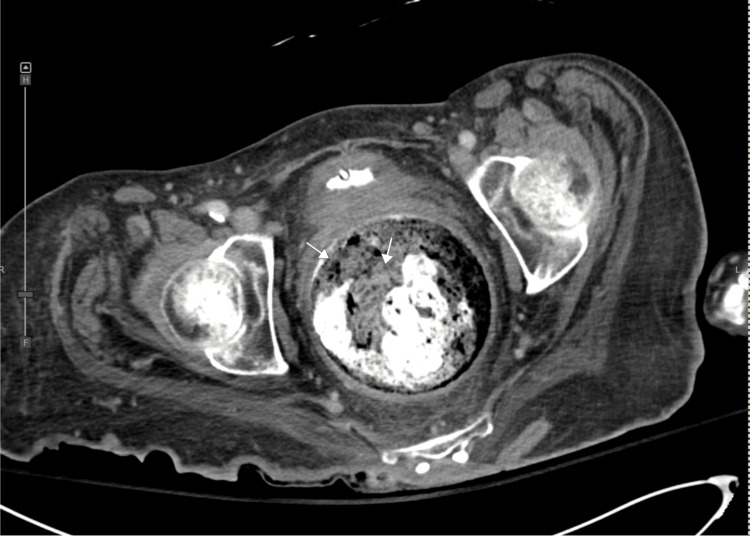
Contrast-Enhanced CT of Abdomen and Pelvis Contrast-enhanced CT scan of the abdomen and pelvis demonstrating stercoral colitis with colonic wall thickening, marked colonic distension, and a significant stool burden.

Subsequently, a CT scan of the abdomen and pelvis was ordered, which indicated findings consistent with stercoral colitis and fecal impaction, characterized by a significant colorectal stool burden (Figure 2).

In the absence of soft tissue infections, *Clostridium perfringens* bacteremia was attributed to translocation secondary to stercoral colitis. She was treated with a seven-day course of intravenous metronidazole, chosen for its effectiveness against gram-positive anaerobes. Stercoral colitis was managed with an aggressive bowel regimen with laxatives and enemas. 

Throughout her hospital stay, she remained hemodynamically stable without evidence of sepsis or other complications. She was discharged in stable condition after completing antibiotics, with no signs of sepsis or recurrent infection. Her condition was optimized for outpatient management, with follow-up arranged with neurology for Parkinson's disease and gastroenterology for an outpatient colonoscopy to rule out any underlying malignancy. The rare presentation of *Clostridium perfringens* bacteremia secondary to stercoral colitis highlights the importance of considering gastrointestinal sources in atypical bacteremia cases, a finding rarely reported in the literature.

## Discussion

Stercoral colitis (SC) is an uncommon but serious complication of chronic constipation, usually affecting elderly or neurologically impaired patients. Prolonged fecal impaction can result in colonic distension, ischemic necrosis, and, in severe cases, perforation, which carries mortality rates of 32-60% [5,7]. Computed tomography is the diagnostic modality of choice and often reveals fecalomas, colonic dilation, and inflammatory fat stranding [8].

Our patient’s background of advanced Parkinson’s disease, dementia, and immobility placed her at high risk for SC. The distinctive feature of this case was the presence of *Clostridium perfringens* bacteremia. The organism’s virulence is related to toxin production [9], often leading to rapidly progressive soft tissue or intra-abdominal infections. In this case, however, there was no clinical or radiographic evidence of gas gangrene, cellulitis, or perforation. Instead, bacterial translocation across an inflamed, compromised colonic wall secondary to increased mucosal permeability from SC was the most plausible source of bacteremia [6].

To our knowledge, only limited case reports have described *Clostridium perfringens* bacteremia occurring in the setting of stercoral colitis or severe constipation. One report described a fatal outcome associated with dual clostridial bacteremia (*C. perfringens* and *C. ramosum*) [10], while another reported fulminant *C. perfringens* sepsis attributed to presumed gastrointestinal translocation in the context of severe constipation and stercoral colitis [11]. Early recognition, metronidazole therapy, and aggressive bowel management (laxatives, enemas) likely contributed to her survival. Management of SC requires urgent bowel decompression, hydration, and avoidance of constipating medications [12]. Manual or endoscopic disimpaction, enemas, and laxatives are often necessary, while surgical consultation is warranted when ischemia, necrosis, or perforation is suspected [12]. In our patient, the addition of PEG tube placement addressed the underlying nutritional compromise, further reducing recurrence risk.

This case highlights stercoral colitis as a potential gastrointestinal source of *C. perfringens* bacteremia, even in the absence of perforation or soft tissue involvement. Awareness of this association may aid early diagnosis and improve outcomes in high-risk patients.

## Conclusions

This case illustrates a rare instance of isolated *Clostridium perfringens* bacteremia secondary to stercoral colitis in a patient with advanced Parkinson's disease, without soft-tissue infection or bowel perforation. Despite the serious nature of this infection, our patient achieved a favorable outcome through early recognition, prompt targeted antimicrobial therapy, and aggressive bowel management. This report expands understanding of atypical *C. perfringens* presentations and underscores stercoral colitis as a potential gastrointestinal source of gram-positive bacteremia in this context. Healthcare providers should maintain a high clinical suspicion for stercoral colitis in neurologically impaired patients with constipation and consider it a source of atypical bacteremia, even when classic associations or manifestations are absent. Early intervention with targeted antibiotics, bowel decompression, and preventive measures such as nutritional optimization may significantly improve outcomes in such challenging cases.
